# Anti‐Lipogenic Effects of Iridoid‐Rich *Lonicera caerulea* Berry Extracts in a Cortisol‐Stimulated Adipocyte Model

**DOI:** 10.1111/1750-3841.71012

**Published:** 2026-03-28

**Authors:** Liangchuan Guo, Yingjun Cui, Damith Costa, Jinli Qiao, Junwei Huo, H. P. Vasantha Rupasinghe

**Affiliations:** ^1^ College of Horticulture and Landscape Architecture Northeast Agricultural University Harbin China; ^2^ College of Life Science Northeast Agricultural University Harbin China; ^3^ Department of Plant, Food, and Environmental Sciences, Faculty of Agriculture Dalhousie University Truro Nova Scotia Canada; ^4^ Department of Pathology, Faculty of Medicine Dalhousie University Halifax Nova Scotia Canada

**Keywords:** 3T3‐L1 cells, functional food, haskap berry, lipid metabolism, oxidative stress, phytonutrients

## Abstract

Berries of *Lonicera caerulea* (blue honeysuckle; haskap) have been used since ancient times in traditional medicine in China, Japan, and northern Russia. This study aimed to extract, characterize, and evaluate the biological activity of iridoid‐rich fractions from haskap berries using an in vitro adipocyte model. Liquid chromatography‐mass spectrometry (UPLC–MS) analysis was performed to quantify the major iridoid distribution among ten haskap cultivars. Two iridoid‐enriched extracts were prepared via ultrasonication‐assisted ethanolic extraction followed by C_18_ solid‐phase fractionation (Extract 1) and subsequent iridoid concentration (Extract 2). Functional evaluation was carried out using a cortisol‐induced 3T3‐L1 adipocyte model. UPLC‐MS revealed pronounced genotypic variation in iridoid profiles among ten cultivars, with CBS‐5 exhibiting the highest total iridoid content (2.42 mg/g dry weight), dominated by loganic acid. UPLC–MS confirmed loganin and loganic acid as the predominant constituents in both extracts. The iridoid‐rich extracts significantly attenuated cortisol‐induced lipid accumulation and intracellular reactive oxygen species generation in 3T3‐L1 adipocytes. Consistently, authentic standards of loganin and loganic acid exhibited pronounced antioxidant and anti‐lipogenic activities. Collectively, these findings indicate that iridoid‐rich extracts from *L. caerulea* berries may modulate lipid dysregulation and oxidative stress, supporting their potential development as functional food ingredients or nutraceuticals.

**Practical Applications:**

Iridoid‐rich haskap berry extracts demonstrate anti‐lipogenic and antioxidant effects in adipocytes, highlighting cultivar selection and extraction strategies for developing functional foods or nutraceutical ingredients targeting stress‐related lipid dysregulation and metabolic health, with potential applications in disease‐prevention formulations and ingredient standardization.

## 1 Introduction

Haskap berry (*Lonicera caerulea*), also called blue honeysuckle, has a long history of use in traditional medicine in China, Japan, and northern Russia, where it has been valued for the management of various chronic disorders (Rupasinghe et al. 2012). This ethnomedicinal use has stimulated contemporary scientific interest in haskap berry as a functional food and source of bioactive compounds for chronic disease prevention and management. The haskap berry is a rich source of plant iridoids (Oszmiański and Kucharska 2018). Iridoids are a class of plant secondary metabolites classified as heterocyclic monoterpenoids, characterized by a cyclopentane‐fused pyran ring structure. The cyclopentane ring forms the core scaffold of iridoids. Cleavage between carbon atoms C‐7 and C‐8 of the cyclopentane ring leads to the formation of secoiridoids, while cleavage of the pyran ring gives rise to various iridoid derivatives (Przybylska et al. 2023). Iridoids contribute to plant responses to both biotic and abiotic stresses. Iridoids are widely distributed among various plant families, including Scrophulariaceae, Pyrolaceae, Oleaceae, Lamiaceae, Rubiaceae, Caprifoliaceae, and Gentianaceae (Hernández Lozada et al. 2022, Kucharska et al. 2017, Wang et al. 2020). Among fifteen iridoids identified in haskap berries, loganic acid was the predominant compound, accounting for 22% to 73% of the total quantified iridoids (Kucharska et al. 2017, Costa and Rupasinghe 2025, Kucharska and Fecka 2016). Solvent‐based extraction with sonication or reflux is commonly used to isolate iridoids from plant matrices (Oszmiański and Kucharska 2018, Kucharska and Fecka 2016).

Among many reported biological properties, iridoids attenuate lipid dysmetabolism both in vivo and in vitro. Androsin, a secoiridoid glycoside, reduced hepatic lipid accumulation in a high‐fat‐high‐fructose diet‐induced non‐alcoholic fatty liver disease (NAFLD) model (Singh et al. 2024). Asperuloside, a cyclopentanoid iridoid glycoside, alleviated lipid deposition in obese mouse livers and HepG2 cells through enhancing lipolysis and suppressing de novo lipogenesis (He et al. 2024). Supplementation of oleuropein, another secoiridoid glycoside, modulated lipogenesis and decreased body weight, adipose tissue mass, and hepatic triglyceride levels in an NAFLD rat model (Hadrich et al. 2016, Sotoudeheian et al. 2023). An oleuropein‐enriched extract from *Jasminum grandiflorum* L. flowers reduced body and liver weights and mitigated lipid dysmetabolism and hepatic steatosis in high‐fat diet‐fed mice and oleic acid‐induced AML‐12 hepatocytes (Hou et al. 2023). Oleuropein also regulated lipid oxidation and lipogenic pathways (Zhou and Du 2024). Loganin improved dyslipidemia in db/db mice (Yamabe et al. 2010). Gentiopicroside decreased intracellular lipid droplet accumulation and triglyceride content in 3T3‐L1 adipocytes (Choi et al. 2019) and alleviated lipid disorders by inhibiting lipogenesis (Ding et al. 2025).

Iridoids have demonstrated potential in protecting cells from oxidative damage and play an inherent role in defending against infections caused by microorganisms, as well as in facilitating the rapid repair of damaged tissues. Iridoid glycosides derived from various medicinal plants have exhibited therapeutic effects in the treatment of several diseases, including neurological disorders, diabetes mellitus, cardiovascular diseases, and cancers (Przybylska et al. 2023, Wang et al. 2020, Chan et al. 2020, Kim and Choi 2021, Marchetti et al. 2024, Tenuta et al. 2020).

Despite the recognized biological activities of iridoids, their composition in haskap berries and effects on adipocyte lipid metabolism remain underexplored. Cortisol is one of the biomarkers of hypothalamic‐pituitary‐adrenal (HPA) axis hyperactivity in response to a stressor and has been demonstrated in many individuals with depression (Murphy et al. 2022). Cortisol can also stimulate preadipocyte differentiation (Marcolongo et al. 2008, Priyadarshini et al. 2018). In this study, we used the cortisol‐stimulated cell model to imitate stress‐related lipid dysregulation. We quantified major iridoids in ten blue honeysuckle cultivars, prepared iridoid‐rich extracts, and evaluated their effects on cell viability, lipid accumulation, and oxidative stress in cortisol‐induced 3T3‐L1 adipocytes.

## 2 Materials and Methods

### 2.1 Samples

Nine haskap berry cultivars (‘CBS‐5’, ‘CBS‐2’, ‘Lanjingling’, ‘Berel’, ‘Wulan’, ‘A1’, ‘RB’, ‘P4‐9’, and ‘P3‐20’) were collected from the Small Berry Germplasm Resource Garden at Northeast Agricultural University, Harbin, China. ‘Lanjingling’ and ‘Wulan’ were developed through hybrid breeding, while the other cultivars were candidate lines obtained from introductions and seed propagation (Zhu et al. 2022). In addition, the cultivar ‘Aurora’ was obtained from Atlantic Haskap Farm in Elderbank, Nova Scotia, Canada. Fully matured berries were used in all experiments.

### 2.2 Extraction of Iridoids From Haskap Cultivars

Frozen haskap berries were lyophilized, ground into a fine powder, and stored at −20°C. Iridoids were extracted from 0.5 g of powder with 10 mL of 60% ethanol (1:20, w/v) using ultrasonication‐assisted extraction for 20 min at room temperature. The extracts were centrifuged and filtered through a 0.22 µm nylon membrane before ultra‐performance liquid chromatography‐electrospray ionization‐mass spectrometry (UPLC–ESI–MS) analysis.

### 2.3 Preparation of Iridoid‐Rich Extracts

Fifty grams of freeze‐dried haskap berry ‘Aurora’ powder were extracted with 1 L of 60% ethanol by ultrasonication for 20 min at room temperature. A previously described flash chromatography using a C_18_ brominated styrenic adsorbent (particle size 250 mm, surface area 630 m^2^/g; Sorbent SP207‐05 Sepabeads resin Sorbent Technologies, Atlanta, GA, USA) was used to purify iridoids (Rupasinghe et al. 2019). Briefly, the 100 mL of filtered crude extract was diluted to 150 mL with 60% ethanol and loaded onto the column, which was equilibrated with 500 mL of 60% ethanol. The column was first washed with 3 L of water to remove impurities such as sugars, followed by stepwise elution with 400 mL of 20%, 40%, 60%, 80%, and 1.2 L of 100% ethanol. Fractions (200 mL each) were collected, and iridoids were mainly detected in the second to sixth fractions of the 100% ethanol eluates. These fractions, which showed the highest iridoid content, were pooled and concentrated under reduced pressure (<50°C) using a rotary evaporator. The concentrated extract was freeze‐dried to yield Extract 1. Considering that anthocyanins are chemically unstable under alkaline conditions (Khoo et al. 2017), a portion of Extract 1 was mixed with six volumes of 0.5 N NaOH to facilitate the degradation and removal of anthocyanins, thereby further enriching the iridoids. The resulting solution was subsequently freeze‐dried to obtain Extract 2.

### 2.4 Characterization of the Iridoid‐Rich Extracts

#### 2.4.1 Total Anthocyanin Content (TAC)

TAC was determined using the pH‐differential spectrophotometric method and expressed as mg cyanidin‐3‐*O*‐glucoside equivalents per g dry weight (mg C3GE/g DW) (ε = 26,900 L·mol^−^^1^·cm^−^^1^; MW = 449.2 g·mol^−^^1^) (Rupasinghe et al. 2015).

#### 2.4.2 Total Phenolic Content (TPC)

TPC was measured by a modified Folin–Ciocalteu method. Concentrations were calculated using a gallic acid standard curve and expressed as mg gallic acid equivalents per g DW (mg GAE/ g DW) (Rupasinghe et al. 2015).

#### 2.4.3 The Total Flavonoid Content (TFC)

TFC was quantified using the aluminum chloride colorimetric assay. Concentrations were calculated against a catechin standard curve and expressed as mg catechin equivalents per g DW (mg CE/g DW) (Rupasinghe et al. 2015).

#### 2.4.4 Quantification of Iridoids by Ultra‐High‐Performance Liquid Chromatography‐Electrospray Ionization‐Mass Spectrometry (UPLC‐ESI‐MS)

Quantification of iridoids was performed in triplicate using UPLC‐ESI‐MS (Waters Acquity UPLC‐SQD‐2, Waters Corporation, Milford, MA, USA) (Costa and Rupasinghe 2025). Briefly, an Acquity BEH C_18_ (100 mm × 2.1 mm, 1.7 µm) column (Waters, Milford, MA, USA) with a flow rate of 0.3 mL/min gradient mobile phase using 0.1% formic acid in water (A) and 0.1% formic acid in acetonitrile (B): Solvent B applied at time t (min); (t, B%): (0, 6%), (6, 20%), (8, 80%), (9, 80%), (10, 6%) was used for the separation of analytes. The MS system was operated in negative ion mode (ESI‐), with a capillary voltage of 3000 V and a nebulizer gas (N_2_) temperature of 375°C. The analytes were identified using single ion mode (SIM): *m/z* 435.1 for loganin, *m/z* 375.1 for loganic acid, and *m/z* 403.1 for sweroside. For quantification, calibration curves were generated using external standards (purity >98%). The limit of detection was between 0.01 and 0.1 mg/L.

#### 2.4.5 Determination of Total Antioxidant Capacity

The DPPH radical scavenging activity was determined using the 2,2‐diphenyl‐1‐picrylhydrazyl (DPPH) assay. Antioxidant capacity was calculated based on a Trolox standard curve and expressed as mg Trolox equivalents per g DW (mg TE/g DW) (Costa and Rupasinghe 2025). The ABTS radical cation scavenging activity was measured using the 2,2′‐azino‐bis (3‐ethylbenzothiazoline‐6‐sulfonic acid) assay. Results were calculated against a Trolox standard curve and expressed as mg Trolox equivalents per g DW (mg TE/g DW) (De Silva and Rupasinghe 2021). Ferric reducing antioxidant power (FRAP) was assessed according to the FRAP assay. The reducing capacity was calculated using an FeSO_4_·7H_2_O standard curve and expressed as mg Fe^2^^+^ equivalents per g DW (mg FeSO_4_·7H_2_O/g DW) (Costa and Rupasinghe 2025).

### 2.5 Cell Culture

Preadipocytes (3T3‐L1 cells, CL‐173, ATCC, Manassas, VA, USA) were maintained in T75 cm^2^ culture flasks in high‐glucose Dulbecco's Modified Eagle's Medium (DMEM) (4.5 g/L D‐glucose) containing 10% fetal bovine serum (FBS) and antibiotics, at 37°C in a humidified 5% CO_2_ incubator. Cells used in the experiments were limited to passage number 10.

### 2.6 Differentiation Protocol

Mouse preadipocyte 3T3‐L1 cells (ATCC CL‐173) were seeded into 96‐well plates. After three days, the cells were induced to differentiate (designated as day 0) using a hormonal differentiation medium consisting of DMEM supplemented with 10% FBS, 1 µM cortisol, 500 µM 3‐isobutyl‐1‐methylxanthine (IBMX), 1 µg/mL insulin, and antibiotics. On day two, the medium was replaced with insulin medium containing DMEM with 1 µg/mL insulin and 10% FBS. Cells were maintained in this medium for the next four days, with medium changes every other day. For evaluating the biological effects, the iridoid‐rich extracts and reference pure iridoids (loganin and loganic acid) (final vehicle concentration 0.1% DMSO) were added together with the differentiation medium on day zero. The doses of the two tested extracts were calculated to consist of 1, 10, or 50 µg of total iridoids per mL of cell culture. On day two, the medium was replaced with insulin medium. The medium was refreshed with insulin medium on day four. On day six, cells were collected for analysis. All assays were performed on day six.

### 2.7 Cell Viability Assay

Cell viability was assessed using the MTS assay (Arumuggam et al. 2017). Briefly, cells were seeded at a density of 5 × 10^3^ cells/well in 96‐well plates and incubated at 37°C in a humidified 5% CO_2_ incubator. Cells were treated with test compounds for 24 or 48 h. Then, 10 µL of a solution containing the MTS reagent (3‐(4,5‐dimethylthiazol‐2‐yl)‐5‐(3‐carboxymethoxyphenyl)‐2‐(4‐sulfophenyl)‐2H‐tetrazolium, inner salt) and the electron coupling reagent phenazine methosulfate (PMS) in a 20:1 ratio was added to each well and incubated for 3 h. Optical density (OD) was measured at 490 nm using a microplate reader. Cell viability was expressed as a percentage relative to the negative control (set to 100%).

### 2.8 Oil Red O (ORO) Staining Assay

3T3‐L1 preadipocytes were seeded at a density of 1 × 10^4^ cells/well in 96‐well plates (Sekhon‐Loodu and Rupasinghe 2019). Cells were treated with or without test compounds and differentiated as described above. On day six, cells were washed twice with PBS, fixed with 4% paraformaldehyde for 45 min in the dark, and stained with 3 mg/mL Oil Red O in 60% isopropanol for 15 min at room temperature. Excess dye was removed by rinsing with PBS. Stained cells were observed under a phase‐contrast microscope at 100× magnification. Lipid droplets were eluted using 100% isopropanol, and absorbance was measured at 492 nm using a microplate reader. Lipid accumulation was expressed as a percentage increase in Oil Red O retention relative to the untreated control.

### 2.9 2′, 7′‐Dichlorofluorescin Diacetate (DCFDA) Assay

3T3‐L1 preadipocytes were seeded at a density of 1 × 10^4^ cells/well in a black, clear‐bottomed 96‐well plate. Cells were treated with or without test compounds and differentiated as described. On day six, cells were washed twice with PBS and incubated with 200 µL of 10 µM DCFDA diluted in clear high‐glucose DMEM for 40 min in the dark under standard culture conditions. After removing the DCFDA solution, clear high‐glucose DMEM‐containing 10% FBS was added. The cells were incubated for 10 min under standard culture conditions. Fluorescence intensity was measured at 485/535 nm (excitation/emission) using a fluorescence plate reader.

### 2.10 Statistical Analysis

Data are presented as mean ± standard deviation (SD) or mean ± standard error of the mean (SEM) from at least three independent experiments, as indicated in the figure legends. Statistical analyses were performed using GraphPad Prism 8 (GraphPad Software, Boston, MA, USA). Differences among groups were evaluated by one‐way analysis of variance (ANOVA) followed by Tukey's post hoc test. Statistical significance was considered at *P* < 0.05 using Minitab (Minitab, LLC).

## 3 Results

### 3.1 Quantitative Analysis of Iridoids in Berries of Haskap Berry Cultivars

**FIGURE 1:**
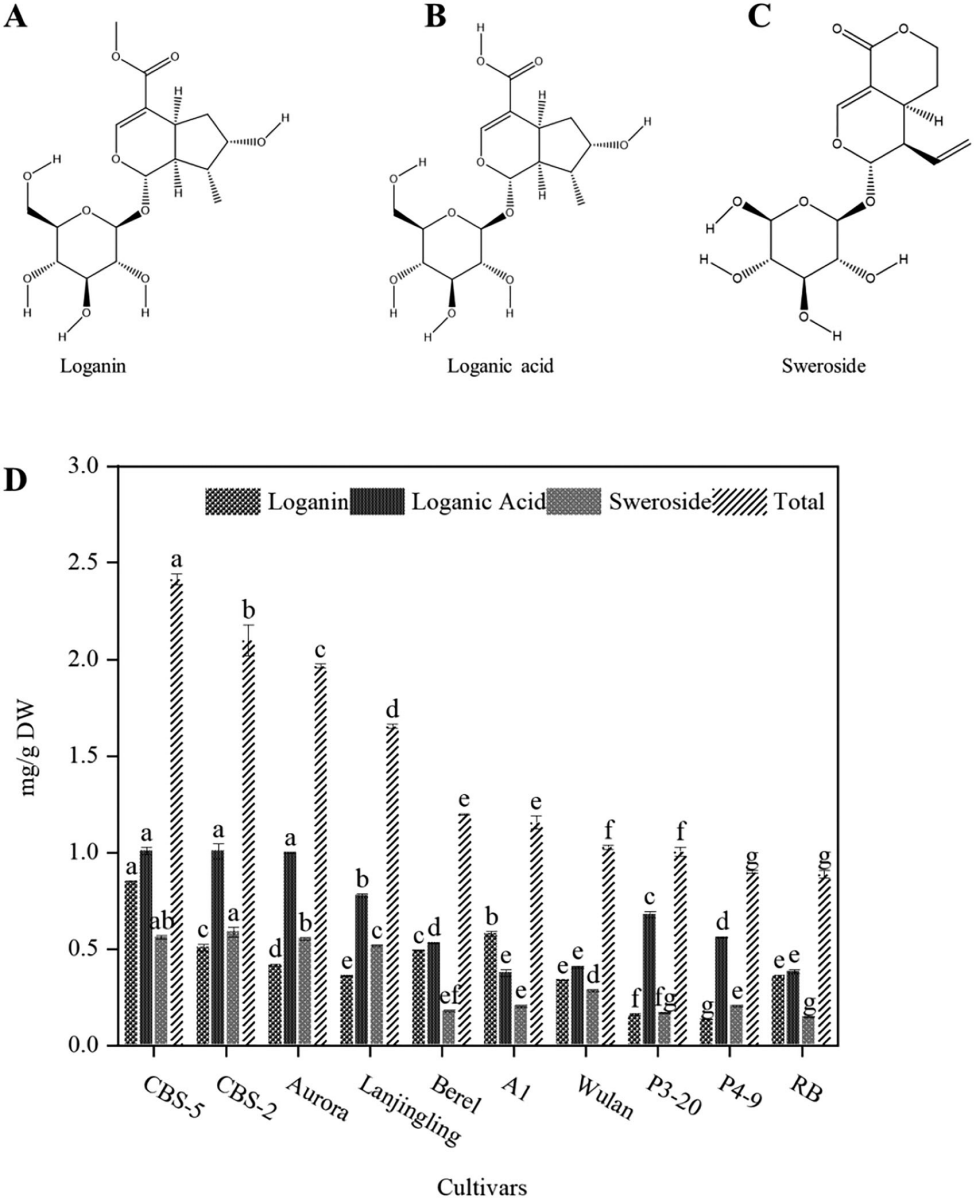
Contents of three major iridoids and total iridoids in berries of ten *Lonicera caerulea* cultivars. Values are expressed as mean ± SEM (*n* = 3). Chemical structures of loganin (A), loganic acid (B), and sweroside (C) and their concentrations in 10 cultivars of haskap berry (D). Different lowercase letters (a—g) above the bars indicate significant differences among the cultivars for each compound (*P* < 0.05).

The contents of loganin, loganic acid, and sweroside varied significantly among the ten cultivars (Figure 1A‐D). Cultivar CBS‐5 showed the highest total iridoid content (2.42 ± 0.08 mg/g DW), followed by CBS‐2 (2.10 ± 0.06 mg/g DW) and Aurora (1.97 ± 0.07 mg/g DW), whereas cultivars P4‐9 and RB exhibited the lowest (< 0.90 mg/g DW). Loganic acid was the most predominant component across all cultivars, while sweroside remained the lowest. One‐way ANOVA indicated significant differences among cultivars for individual and total iridoids (*P* < 0.05), highlighting genetic variation in iridoid accumulation.

### 3.2 Comparative Characterization of Extract 1 and Extract 2

**FIGURE 2:**
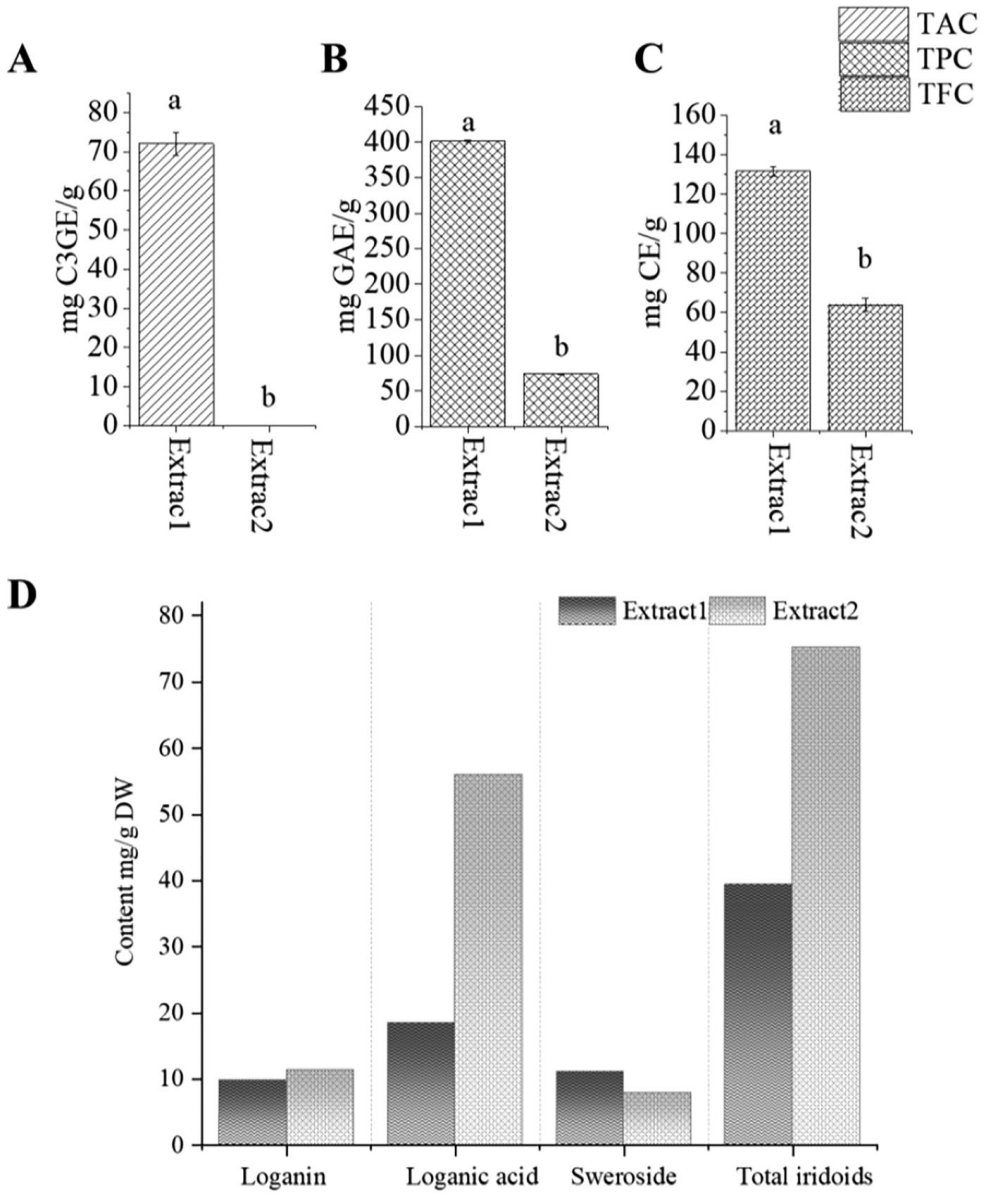
Total anthocyanin content (A), total phenolic content (B), total flavonoid content (C), and loganin, loganic acid, sweroside, and total iridoid concentrations (D) of extract 1 and extract 2 of haskap berry (*n* = 3). Different lowercase letters (a, b) above the bars indicate significant differences among groups (*P* < 0.05). Values are expressed as mean ± SEM (*n* = 3).

**FIGURE 3:**
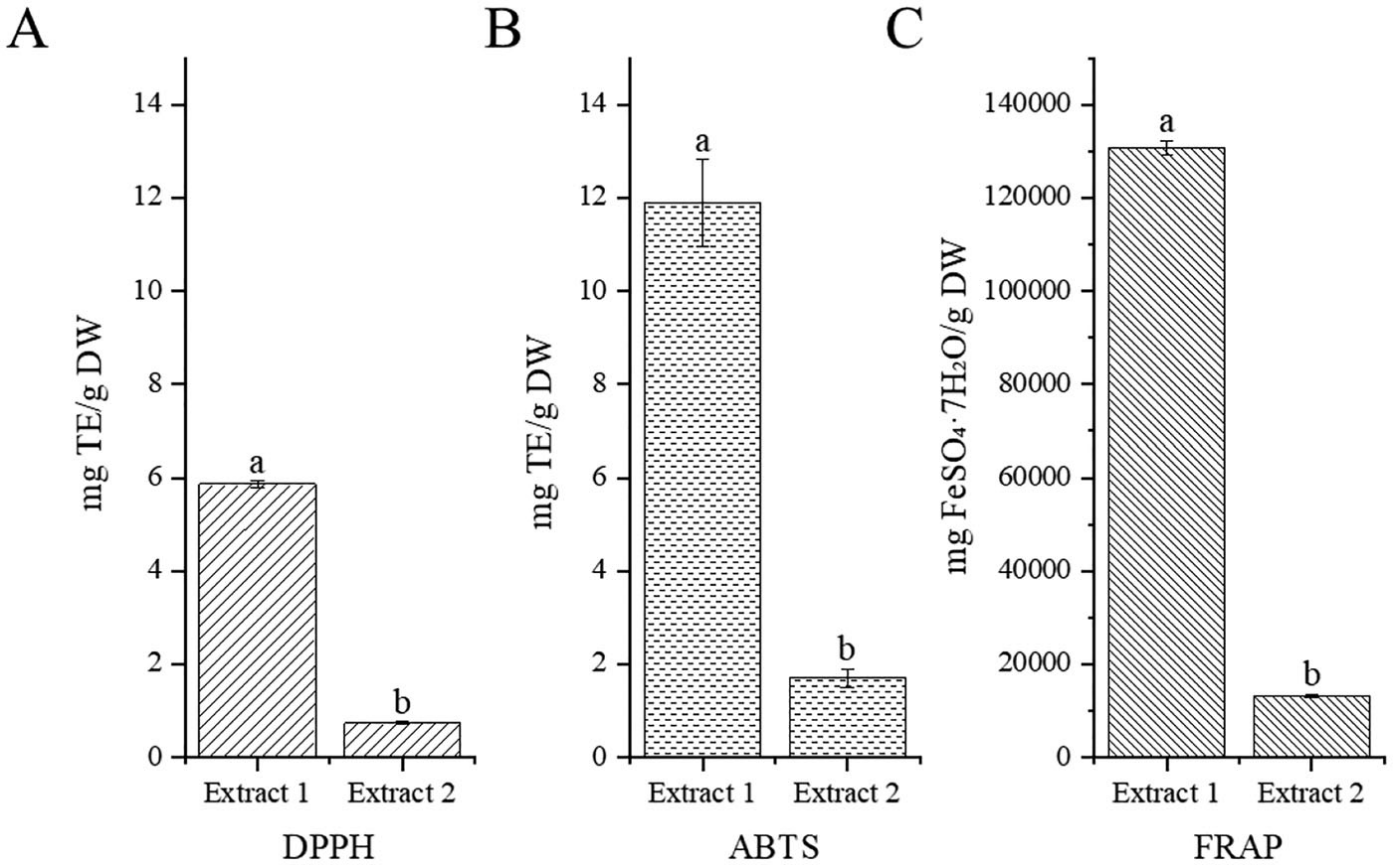
Total antioxidant capacity of extract 1 and extract 2 of haskap berry. A, DPPH radical scavenging activity; B, ABTS radical cation scavenging activity; and C, ferric reducing antioxidant power (FRAP). Trolox was used as the standard for DPPH and ABTS assays, while FeSO_4_·7H_2_O was used as the standard for the FRAP assay. Data are expressed as mean ± SEM (*n* = 3). Different lowercase letters (a, b) above the bars indicate significant differences between extracts (*P* < 0.05).

Significant differences were observed in TAC, TPC, and TFC between the two extracts (Figure 2A–C). Extract 1 contained substantially higher levels of TAC, TPC, and TFC compared with Extract 2. Specifically, the TAC of Extract 1 reached 72.0 ± 5.11 mg/g, whereas Extract 2 contained only trace amounts (0.044 ± 0.01 mg/g). A similar trend was found for total phenolics and total flavonoids: the TPC of Extract 1 was 401 ± 2.49 mg/g, which was more than fivefold higher than that of Extract 2 (73.6 ± 0.44 mg/g), and its TFC reached 132 ± 4.06 mg CE/g, in contrast to 63.8 ± 5.89 mg CE/g in Extract 2. Statistical analysis confirmed that all three indices were significantly higher in Extract 1 than in Extract 2 (*p* < 0.05). These results indicate that the purification and alkaline post‐treatment steps used to obtain Extract 2 effectively removed a large portion of polyphenol‐type co‐extractives, resulting in a more iridoid‐focused chemical profile. These results indicate that extract 1 possesses a substantially greater overall antioxidant potential than extract 2 (Figure 3).

The concentrations of three major iridoids, loganin, loganic acid, and sweroside, differed markedly between Extract 1 and Extract 2 (Figure 2D). Extract 2 exhibited a substantially higher total iridoid content (75.3 mg/g) than Extract 1 (39.4 mg/g), indicating enrichment of iridoids through the removal of co‐eluted polyphenols, especially anthocyanins. Notably, loganic acid and loganin were present at markedly higher levels in Extract 2. Loganic acid was the predominant constituent in both extracts, accounting for over 70% of the total iridoids in Extract 2. Although Extract 1 contained relatively higher proportions of sweroside, its overall iridoid yield was significantly lower (Figure 2D).

### 3.3 Effects of Extracts and Tested Compounds on the Viability of 3T3‐L1 Preadipocytes

**FIGURE 4:**
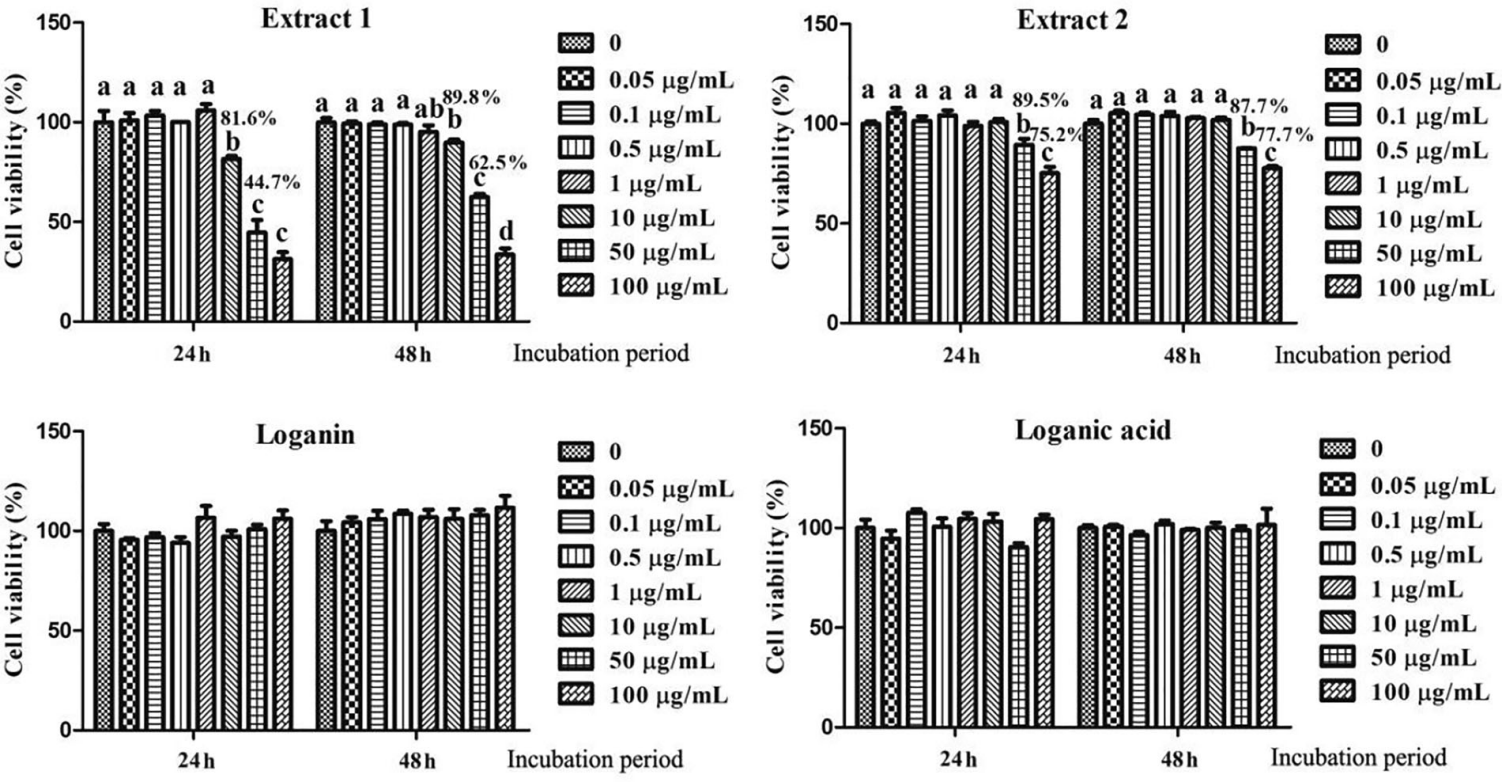
Effects of iridoid‐rich haskap berry extracts, loganin, and loganic acid on the viability of 3T3‐L1 preadipocytes as determined by the MTS assay. Cells were treated with various concentrations of the extracts and tested compounds for 24 or 48 h. Data are expressed as mean ± SEM. The data represent three independent experiments (*n* = 9). Bars not sharing the same letter differ significantly (*P* < 0.05).

3T3‐L1 preadipocytes were treated with various concentrations of the test extracts and compounds and incubated for 24 or 48 h. The concentrations of haskap berry Extract 1 and Extract 2 were 0.05, 0.1, 0.5, 1, 10, 50, and 100 µg/mL, selected based on their loganin/loganic acid content. Loganin and loganic acid were tested at the same concentrations. Cell viability was subsequently assessed using the MTS assay. Treatment with 50 µg/mL of Extract 1 markedly reduced cell viability to 44.7% of control at 24 h and to 62.5% at 48 h (*P* < 0.05) (Figure 4). In contrast, 50 µg/mL of Extract 2 led to a more moderate decrease in cell viability, with levels at 89.5% and 87.7% cell viability of control after 24 and 48 h, respectively, (*P* < 0.05). No significant cytotoxic effects were observed in cells treated with loganin or loganic acid at any tested concentrations.

### 3.4 Iridoid‐Rich Extracts Ameliorate Cellular Lipid Contents Induced by Cortisol in 3T3‐L1 Cells

**FIGURE 5:**
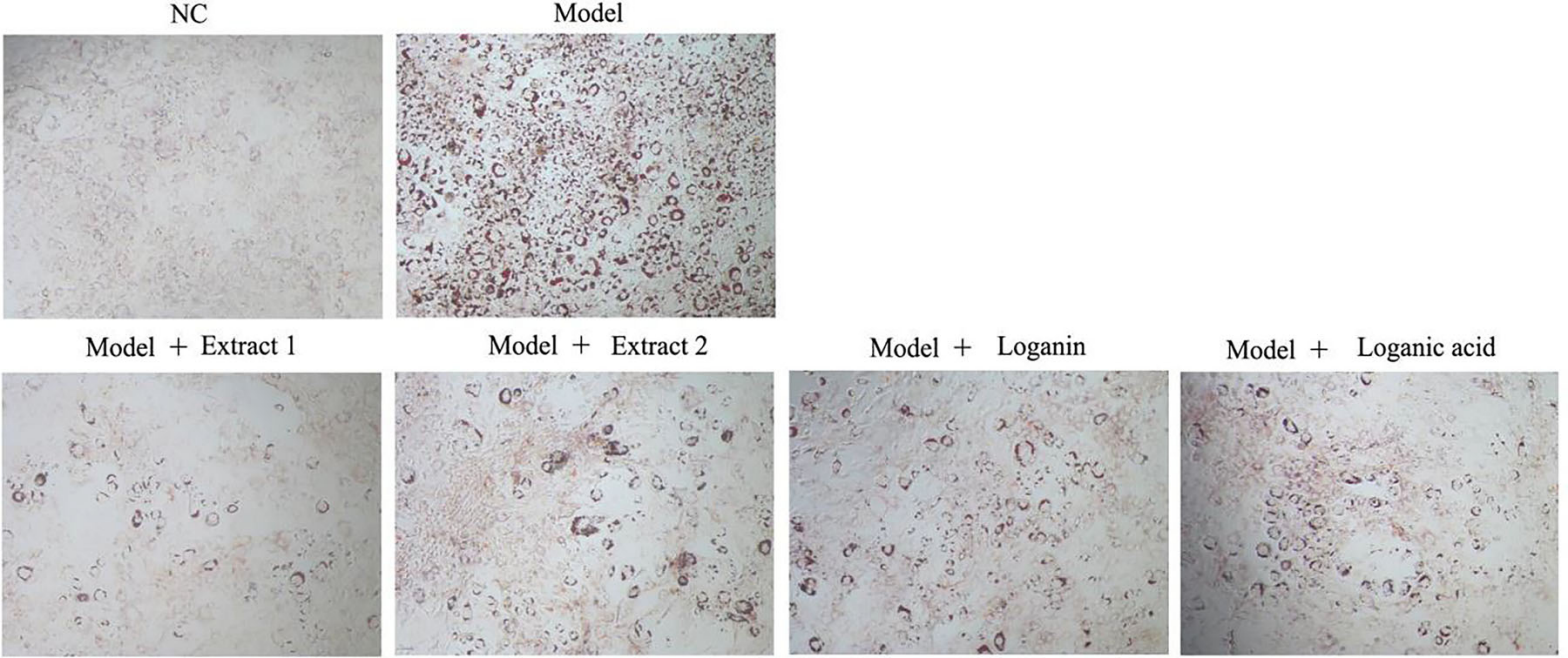
Effects of tested extracts and compounds on cortisol‐induced lipid accumulation in 3T3‐L1 cells. Cells were treated with 1 µM cortisol, 500 µM 3‐isobutyl‐1‐methylxanthine (IBMX), and 1 µg/mL insulin in the presence or absence of the tested compounds for 2 days, followed by insulin treatment (1 µg/mL) for an additional 4 days. Images were acquired following Oil Red O staining under 100× magnification.

**FIGURE 6:**
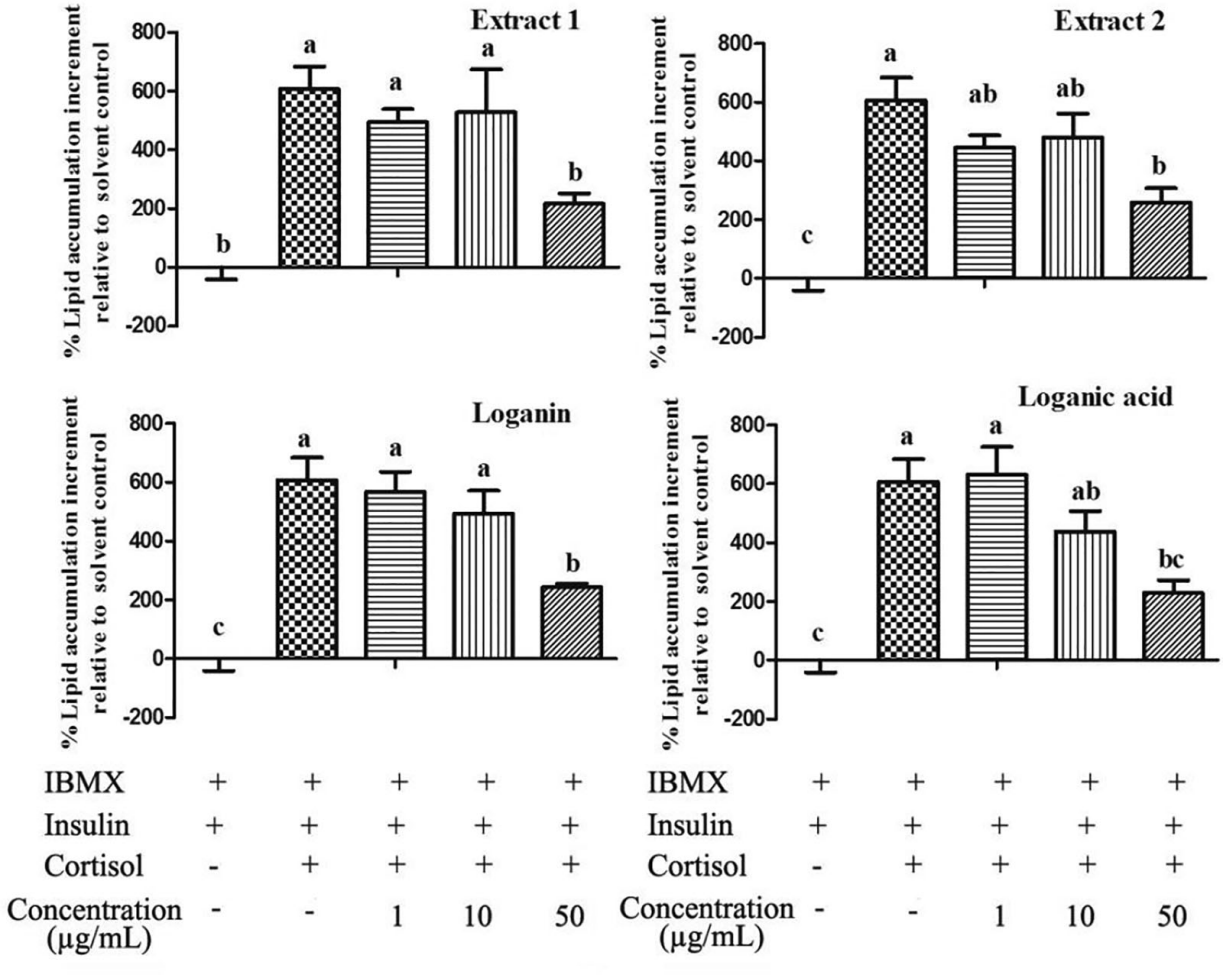
Effects of tested extracts and compounds on cortisol‐induced lipid accumulation in 3T3‐L1 cells as determined by quantitative Oil Red O assay. Cells were treated as described in the methodology. Lipid content was quantified spectrophotometrically following Oil Red O extraction. Data are expressed as mean ± SEM. The data represent three independent experiments (*n* = 9). Bars not sharing the same letter differ significantly (*P* < 0.05).

Oil Red O staining was used to study the effects of the tested extracts and compounds on lipid accumulation in 3T3‐L1 adipocytes. Oil Red O staining revealed a marked reduction in induced‐intracellular lipid content following treatment with 50 µg/mL of haskap berry Extracts 1 and 2, loganin, or loganic acid in 3T3‐L1 cells (Figure 5). Consistently, quantitative analysis using the Oil Red O assay demonstrated that lipid accumulation was significantly reduced by 35.7%, 42.6%, 40.1%, and 37.6%, respectively, from that of the model when treated with the above test compounds (Figure 6) (*P* < 0.05).

### 3.5 Iridoid‐Rich Extracts Reduce Cortisol‐Induced Reactive Oxygen Species (ROS) Production in 3T3‐L1 Cells

**FIGURE 7:**
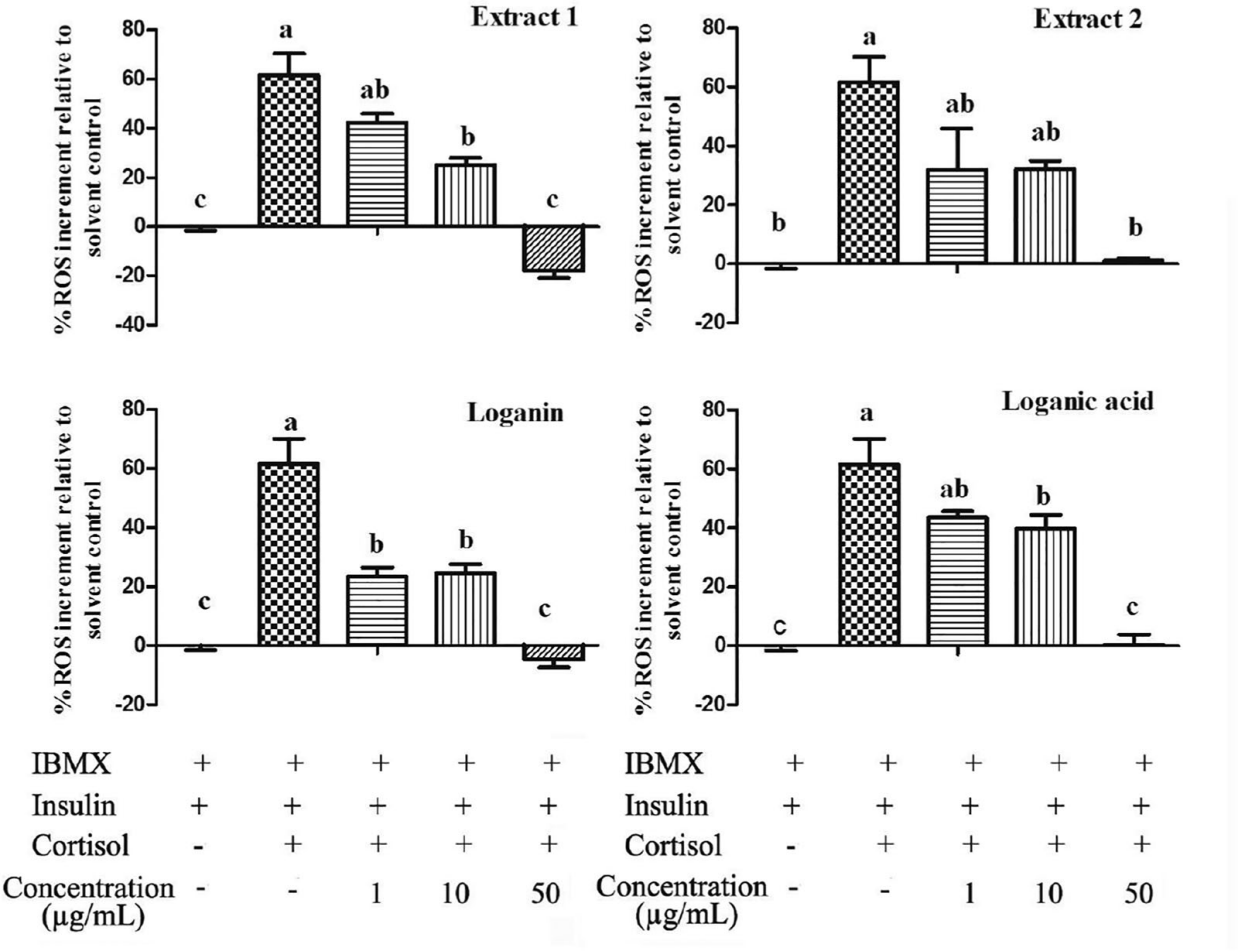
Effects of tested extracts and compounds on cortisol‐induced reactive oxygen species (ROS) production in 3T3‐L1 cells. Cells were treated as described in Figure 5. ROS levels were measured using the DCFDA assay. Data are presented as mean ± SEM. The data represent three independent experiments (*n* = 9). Bars not sharing the same letter differ significantly (*P* < 0.05).

The effects of the tested extracts and compounds on cortisol‐induced ROS production in 3T3‐L1 cells were assessed on day 6. Results from the DCFDA assay demonstrated that treatment with haskap berry Extracts 1 and 2, loganin, and loganic acid significantly and dose‐dependently reduced ROS levels (*P* < 0.05) (Figure 7).

## 4 Discussion

The present study demonstrated substantial genotypic variation in iridoid accumulation among ten haskap berry cultivars, with two new breeding lines (CBS‐5 and CBS‐2), which showed the highest total iridoid content. The observed high variability of iridoids among cultivars agrees with previous reports that secondary metabolite biosynthesis in *Lonicera* species is strongly genotype‐dependent and influenced by both genetic and environmental factors (Guo et al. 2023, Naugžemys et al. 2022). Loganic acid consistently dominated as the most predominant iridoid among cultivars, accounting for more than half of the total iridoid content, corroborating previous findings that this compound is a key chemotaxonomic marker of haskap berry, which is consistent with earlier studies (Oszmiański and Kucharska 2018). The observed differences suggest that certain cultivars have inherently higher biosynthetic capacities for iridoids, likely driven by differential regulation of key enzymes in the monoterpenoid‐derived iridoid biosynthetic pathway (Guo et al. 2025). Consequently, cultivars such as CBS‐5 and CBS‐2 could be used in breeding programs or industrial‐scale extraction to obtain iridoid‐enriched nutraceuticals.

The fractionation strategy developed in this study substantially improved the purity of iridoids in haskap berry extracts. Compared with Extract 1, Extract 2 showed nearly a two‐fold increase in total iridoid content, mainly attributable to the pronounced enrichment of loganic acid and loganin. The downstream purification by removal of sugars and other water‐soluble compounds using C_18_ flash chromatography has increased the iridoid purity and concentration. In particular, Extract 2, derived from Extract 1 through alkaline treatment followed by freeze‐drying, contained a markedly higher iridoid concentration, suggesting that the combined purification and post‐processing procedures effectively enhanced iridoid purity. Additionally, anthocyanins, which are water‐soluble pigments present in haskap berries, are generally unstable under alkaline conditions; their glycosidic bonds can undergo hydrolysis and the loss of positively charged flavylium cation by ring opening. Therefore, the alkaline post‐treatment likely led to the degradation of anthocyanins, further contributing to the enrichment of iridoid in Extract 2 (Khoo et al. 2017, Zhao et al. 2021, Holinesti et al. 2025, Klinger et al. 2025, Yang et al. 2025). However, iridoids may exhibit sensitivity to alkaline conditions. Under strong basic environments, ester bonds can undergo saponification, and glycosidic linkages may be partially hydrolyzed, potentially leading to structural transformation. Previous studies have reported that certain iridoid glycosides undergo hydrolysis under strong alkaline conditions. Therefore, although NaOH treatment effectively minimized anthocyanin interference, possible structural modification of iridoids cannot be completely excluded (Ma et al. 2022). This compositional divergence can be clearly seen when the contents of loganic acid and loganin were compared between Extract 1 and Extract 2 (Figure 2D).

Assessment of cell viability revealed that haskap berry Extract 2 exhibited markedly lower cytotoxicity compared to Extract 1, despite its higher iridoid concentration. This finding suggests that non‐iridoid components or matrix effects present in Extract 1 may contribute to cellular stress or metabolic burden at elevated concentrations, which is consistent with previous studies (Zehfus et al. 2021). However, pure loganin and loganic acid did not impact cell viability at all tested concentrations, which supports the notion that the observed viability reduction in Extract 1‐treated cells arises from co‐extracted phenolic or other compounds (Jeon et al. 2023, Park et al. 2018). The improved safety profile of Extract 2 may be associated with its selective enrichment of iridoids and the concomitant reduction of potentially toxic constituents. Most of these constituents, including high concentrations of anthocyanins, are generally safe at the tested concentrations, but high concentrations could exhibit prooxidant effects or cytotoxicity in certain cell types (Cahyana et al. 2022, Catacutan et al. 2024).

Both iridoid‐rich extracts of haskap berry, as well as the pure loganin and loganic acid, significantly inhibited cortisol‐induced lipid accumulation and ROS production in 3T3‐L1 adipocytes (Figures 5, 6, 7). Oil Red O staining (Figure 5) and quantitative analysis (Figure 6) confirmed a marked reduction in intracellular lipid content following treatment, indicating a potential anti‐adipogenic effect. These findings are consistent with earlier studies reporting that iridoids such as oleuropein, asperuloside, and gentiopicroside exert anti‐lipogenic and antioxidant effects by modulating key regulatory pathways involved in lipid metabolism and redox homeostasis (Park et al. 2018, Park et al. 2020, Park 2013).

Loganin reduces ROS, thereby alleviating oxidative stress and preserving cellular function (Chuang et al. 2025). Iridoids influence lipid and cholesterol metabolism mainly by modulating key transcription factors such as Peroxisome Proliferator‐Activated Receptor Alpha (PPAR‐α), Peroxisome Proliferator‐Activated Receptor Gamma (PPAR‐γ), CCAAT/Enhancer‐Binding Protein Alpha (C/EBPα), Liver X Receptor (LXR), and Sterol Regulatory Element‐Binding Protein 1c (SREBP‐1c), thereby regulating lipid synthesis, storage, and catabolism (Danielewski et al. 2021). Additionally, iridoids from *Valeriana fauriei* promote autophagy, facilitating the breakdown of lipid droplets in hepatocytes (Lee et al. 2020). Consistently, our results demonstrate that iridoid‐rich extracts from haskap berries simultaneously reduce intracellular lipid content and ROS levels (Figure 7), suggesting that these extracts mitigate lipid dysmetabolism partly through alleviating oxidative stress and supporting redox balance (Wang et al. 2023). Both iridoid‐rich extracts showed comparable lipid‐lowering and antioxidant effects, with Extract 2 exhibiting lower cytotoxicity, suggesting its potential for further mechanistic studies and development as a functional ingredient.

However, although the simulated physiological stress–induced adipocyte model yielded novel insights into the anti‐adipogenic potential of the iridoid‐rich haskap berry extracts, several limitations should be acknowledged. First, the complete chemical profile of the extracts remains to be fully characterized; comprehensive phytochemical analysis is required to identify and quantify the bioactive constituents responsible for the observed effects. Future investigations should elucidate the underlying molecular mechanisms by assessing mRNA and protein expression levels of key regulators and markers of adipogenesis, lipogenesis, lipid catabolism, oxidative stress, and inflammation. Quantification of intracellular total triglyceride content would further substantiate the anti‐adipogenic activity. Importantly, validation of these in vitro findings requires well‐designed in vivo studies using appropriate experimental animal models. Such studies should evaluate bioavailability, pharmacokinetics, effective dose ranges, and safety profiles, including potential toxicity. Addressing these aspects will be essential to determine the translational relevance and therapeutic potential of haskap‐derived extracts.

Collectively, these results reveal the dual beneficial effects of *L. caerulea* iridoids in the cortisol‐induced adipocyte model: suppression of lipid accumulation and attenuation of oxidative stress. These preliminary findings provide a scientific basis for further investigation into their molecular targets and potential applications in the prevention or management of metabolic disorders associated with cortisol‐induced adipocyte dysfunction.

## 5 Conclusions

In summary, this study demonstrates that haskap berry is a rich source of bioactive iridoids, with significant genotypic variation among cultivars. Optimized extraction and partial purification strategies yielded iridoid‐rich extracts, with Extract 2 showing higher iridoid content and lower cytotoxicity compared to Extract 1. Both extracts, as well as the authentic loganin and loganic acid, effectively inhibited cortisol‐induced lipid accumulation and reduced ROS in 3T3‐L1 adipocytes. Collectively, these findings suggest the dual lipid‐lowering and antioxidant potential of haskap berry iridoids, suggesting their potential for development as functional ingredients for the prevention or management of metabolic disorders associated with adipocyte dysfunction.

## Author Contributions

**Liangchuan Guo**: conceptualization, investigation, writing – original draft, methodology, visualization, formal analysis. **Yingjun Cui**: conceptualization, investigation, writing – original draft, methodology, visualization, formal analysis. **Damith Costa**: investigation, methodology, writing – review and editing. **Jinli Qiao**: investigation, methodology, writing – review and editing. **Junwei Huo**: conceptualization, funding acquisition, writing – review and editing, methodology, supervision, resources. **H.P. Vasantha Rupasinghe**: conceptualization, investigation, funding acquisition, methodology, project administration, supervision, resources, writing – review and editing.

## Funding

This work was supported by the National Key R&D Program of China (2022YFD1600500) and the Natural Sciences and Engineering Research Council (NSERC) of Canada (RGPIN‐2023‐03324).

## Conflicts of Interest

The authors declare no conflicts of interest.
